# Intractable hiccups as a brainstem red flag: demyelinating disorders with emphasis on neuromyelitis optica spectrum disorder and area postrema syndrome

**DOI:** 10.1007/s10072-026-09288-7

**Published:** 2026-08-15

**Authors:** Sinan Eliaçık

**Affiliations:** Department of Neurology, School of Medicine, Hitit University, Çorum, 19040 Turkey

**Keywords:** Hiccups, Singultus, Neuromyelitis optica spectrum disorder, Area postrema syndrome, Multiple sclerosis, MOG antibody disease, Brainstem demyelination

## Abstract

**Background:**

Persistent and intractable hiccups may indicate brainstem pathology rather than benign gastrointestinal causes. Among inflammatory demyelinating disorders, neuromyelitis optica spectrum disorder (NMOSD) frequently involves the dorsal medulla, particularly the area postrema, a key structure in the hiccup reflex arc. Area postrema syndrome (APS), characterized by otherwise unexplained hiccups, nausea, or vomiting lasting ≥ 48 h, represents a core diagnostic feature of NMOSD.

**Methods:**

A structured narrative literature review was conducted using PubMed, Scopus, and Web of Science databases from inception to January 2026. Studies addressing persistent or intractable hiccups associated with demyelinating disorders, including NMOSD, multiple sclerosis, and myelin oligodendrocyte glycoprotein antibody–associated disease (MOGAD), were analyzed with emphasis on neuroanatomical, immunopathological, and radiological correlations.

**Results:**

NMOSD is the prototypical demyelinating disorder associated with intractable hiccups due to selective astrocytic injury in the area postrema. APS may precede optic neuritis or myelitis and can represent the initial manifestation of disease. Multiple sclerosis and MOGAD may also involve the brainstem but differ in lesion distribution, immunopathology, and treatment implications. Early recognition of dorsal medullary involvement and antibody testing is critical for accurate diagnosis and timely immunotherapy.

**Conclusion:**

Intractable hiccups should be recognized as a potential brainstem red flag symptom. Awareness of their association with NMOSD and related demyelinating disorders facilitates early diagnosis, appropriate immunotherapy, and prevention of neurological disability.

## Introduction

Hiccups (singultus) are defined as involuntary, repetitive contractions of the diaphragm and intercostal musculature followed by abrupt glottic closure, producing the characteristic sound. Episodes are classified as acute (< 48 h), persistent (> 48 h), and intractable (> 1 month). While transient hiccups are benign and common, persistent forms warrant systematic evaluation. Prolonged singultus may lead to significant morbidity including sleep disturbance, nutritional depletion, respiratory muscle fatigue, and rarely aspiration pneumonia, underscoring the need for early etiological investigation [1].

Neurological etiologies are frequently overlooked. Brainstem lesions—particularly within the medulla—can disrupt inhibitory control of the hiccup reflex arc [2]. Over the past two decades, NMOSD has emerged as the most important inflammatory cause of intractable hiccups due to selective involvement of the area postrema. APS is now recognized as a core clinical characteristic in the 2015 International Panel diagnostic criteria for NMOSD [3, 4].

Recent updates have further refined the diagnostic and therapeutic framework of NMOSD, emphasizing early antibody testing and recognition of core clinical characteristics, including APS, even in seronegative cases [5]. Epidemiological analyses suggest that NMOSD remains a rare disorder worldwide, with prevalence estimates generally ranging between 0.5 and 10 per 100,000 individuals depending on geographic region and ethnicity [6]. These data highlight the importance of early recognition of sentinel brainstem symptoms such as persistent hiccups. Although the relationship between APS and NMOSD has been extensively described, previous reviews have primarily focused on APS as a diagnostic criterion of NMOSD. The present review specifically emphasizes persistent and intractable hiccups as an early brainstem warning sign and integrates emerging evidence regarding MRI-negative APS presentations, autonomic manifestations, inflammatory mimics, and evolving therapeutic considerations. Furthermore, practical comparative tables and a clinician-oriented diagnostic algorithm are provided to facilitate differential diagnosis in routine neurological practice.

## Methods

### Search strategy and data sources

This review was conducted using a structured narrative approach with systematic elements. A comprehensive literature search was performed in PubMed, Scopus, and Web of Science from database inception to January 31, 2026.

The primary search was conducted in PubMed using Title/Abstract field restrictions to enhance clinical specificity and yielded 64 records.

The search strategy combined free-text terms and Boolean operators (AND/OR), including:

- “hiccups” OR “singultus”.
- “area postrema syndrome”.
- “neuromyelitis optica” OR “NMOSD”.
- “aquaporin-4 antibody” OR “AQP4-IgG”.
- “myelin oligodendrocyte glycoprotein (MOG)” OR “MOG antibody–associated disease (MOGAD)”.
- “brainstem demyelination”.
- “dorsal medulla”.

**Fig. 1:**
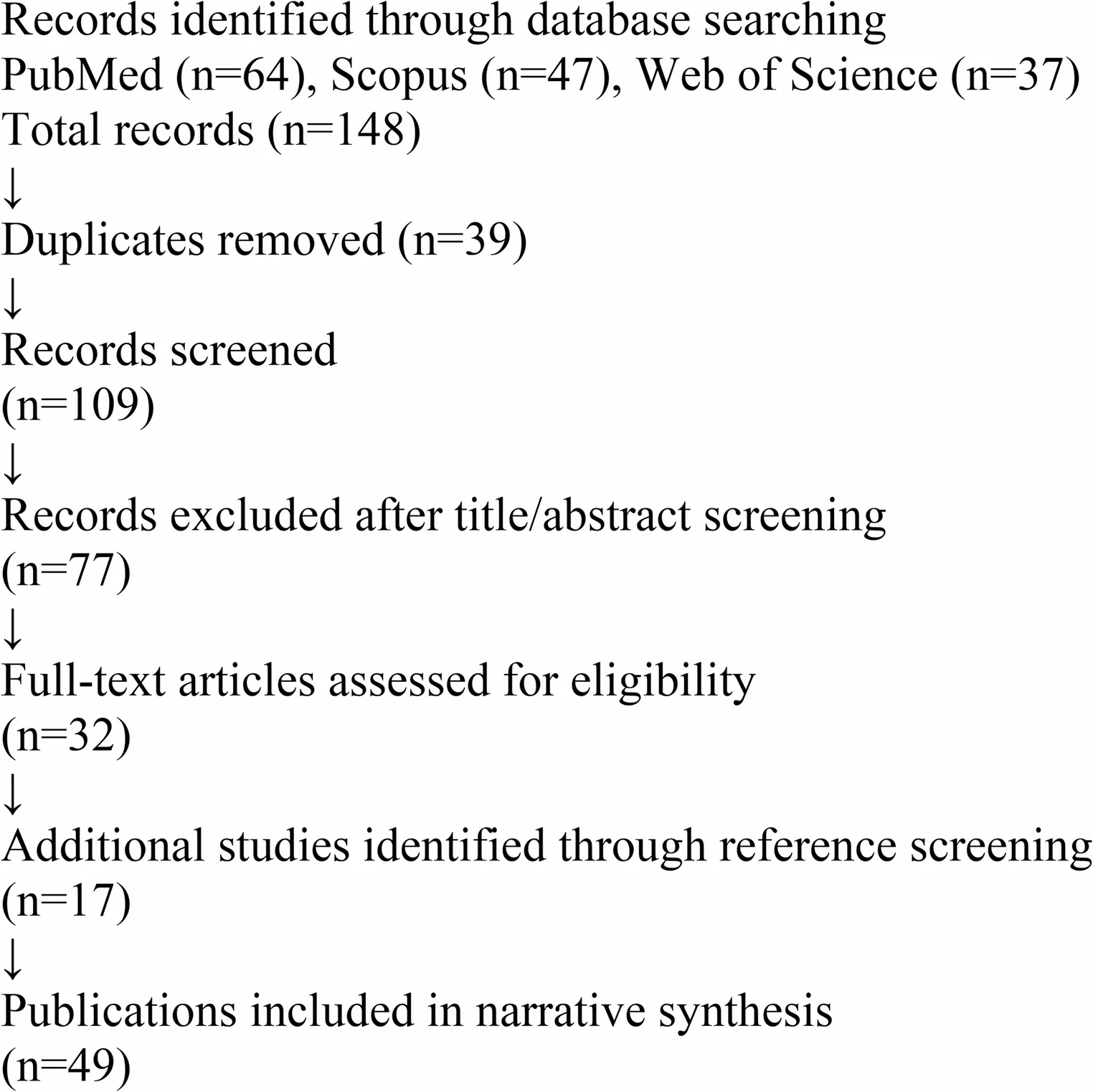
Literature selection flow diagram

Equivalent keyword combinations were adapted for Scopus and Web of Science. Reference lists of relevant articles were manually screened to identify additional eligible studies not captured through database searching. Overall, 148 records were identified across PubMed, Scopus, and Web of Science. After duplicate removal, titles and abstracts were screened according to predefined eligibility criteria. Full-text articles were subsequently assessed, and additional relevant studies were identified through manual review of reference lists. The study selection process is summarized in Fig. 1.

Figure 1. Literature selection flow diagram. A structured narrative review with systematic elements was conducted. A total of 148 records were identified through database searching (PubMed, Scopus, and Web of Science). After removal of 39 duplicate records, 109 articles underwent title and abstract screening. Thirty-two full-text articles were assessed for eligibility, and an additional 17 relevant publications were identified through manual screening of reference lists, resulting in 49 studies included in the final narrative synthesis.

A structured narrative review with systematic elements was performed. Literature searches were conducted in PubMed, Scopus, and Web of Science from database inception to January 31, 2026. Overall, 148 records were identified (PubMed *n* = 64, Scopus *n* = 47, Web of Science *n* = 37). After removal of 39 duplicate records, 109 articles underwent title and abstract screening. Thirty-two full-text articles were assessed for eligibility, and 49 publications were ultimately included in the narrative synthesis. Because of the heterogeneity of study designs and the predominance of case reports and observational studies, a quantitative meta-analysis was not performed.

Unlike previous reviews focusing primarily on APS as a diagnostic criterion, this review emphasizes persistent hiccups as a sentinel inflammatory brainstem signal and integrates emerging data on imaging-negative APS and autonomic instability.

### Eligibility criteria

Studies were eligible if they met one or more of the following criteria:

1. Original clinical studies (prospective or retrospective cohorts)
2. Case series or case reports describing persistent or intractable hiccups associated with central demyelinating disorders
3. Review articles addressing APS in NMOSD or MOGAD
4. English-language publications

Studies were excluded if they:

- Focused exclusively on gastrointestinal, toxic, or metabolic causes of hiccups
- Were conference abstracts without full-text availability
- Lacked sufficient clinical, radiological, or immunological detail

### Study selection and data extraction

Titles and abstracts were screened for relevance based on predefined eligibility criteria. Full-text articles were retrieved and assessed when necessary to confirm inclusion.

Data extraction was performed in a structured manner. The following variables were collected when available:

- Patient demographics
- Antibody status (AQP4-IgG, myelin oligodendrocyte glycoprotein immunoglobulin G (MOG-IgG))
- Clinical presentation, including characteristics of singultus
- Magnetic Resonance Imaging (MRI) findings, particularly dorsal medullary involvement
- Treatment strategies
- Clinical outcomes and relapse patterns

Given the heterogeneity of study designs (predominantly case reports, case series, and small observational cohorts), formal quantitative synthesis or meta-analysis was not undertaken. Key studies included in this review, together with their clinical characteristics, MRI findings, antibody status, and outcomes, are summarized in Table 1.

### Methodological considerations

As this review synthesizes heterogeneous evidence, formal risk-of-bias assessment tools were not systematically applied. However, study design, sample size, peer-review status, and methodological clarity were considered during interpretation. Greater interpretative weight was given to multicenter cohorts and well-characterized immunological studies when available. This review represents a structured narrative synthesis and does not adhere to a formal PRISMA-guided systematic review framework. Prospective protocol registration (e.g., PROSPERO) was not performed, as no quantitative meta-analysis was planned.

**Table 1:** Representative studies included in the present review

Author		Year	Disease	Hiccups/APS Presentation		MRI Findings		Antibody Status			Outcome
Wingerchuk et al.	2006		NMOSD		Intractable hiccups and vomiting		Dorsal medulla lesion		AQP4-IgG positive	Improvement after immunotherapy	
Apiwattanakul et al.	2010		NMOSD		Area postrema syndrome		Area postrema hyperintensity		AQP4-IgG positive	Symptom resolution	
Popescu et al.	2010		NMOSD		Persistent hiccups		Medullary lesion		AQP4-IgG positive	Favorable recovery	
Jarius et al.	2016		NMOSD		Persistent hiccups		Medullary lesion		AQP4-IgG positive	Clinical improvement	
Flanagan et al.	2017		GFAP astrocytopathy		Nausea, vomiting, hiccups		Radial perivascular enhancement		GFAP-IgG positive	Steroid responsive	
Juryńczyk et al.	2017		MOGAD		Hiccups and vomiting		Brainstem involvement		MOG-IgG positive	Favorable outcome	
Pittock et al.	2019		NMOSD		Severe relapsing disease		Medullary lesions		AQP4-IgG positive	Reduced relapse rate with eculizumab	
Yamamura et al.	2019		NMOSD		APS associated relapse		Variable MRI findings		AQP4-IgG positive	Improved with satralizumab	
Cree et al.	2019		NMOSD		Recurrent APS		Brainstem lesions		AQP4-IgG positive	Stabilization with inebilizumab	
Hinson et al.	2020		NMOSD		APS and brainstem syndrome		Area postrema involvement		AQP4-IgG positive	Biomarker correlation	
Jarius et al.	2022		NMOSD		Intractable hiccups		Dorsal medullary lesions		AQP4-IgG positive	Good long-term outcome	
Lehmann-Horn et al.	2023		NMOSD		Acute APS attack		Brainstem lesion		AQP4-IgG positive	Early treatment associated with better recovery	
Schindler et al.	2025		NMOSD/MOGAD		Brainstem manifestations		MRI volumetric changes		AQP4-IgG or MOG-IgG positive	Biomarker association	
Dubey et al.	2019		MOGAD		Brainstem symptoms uncommon; APS not characteristic		Longitudinally extensive myelitis with central gray matter involvement (“H-sign”); occasional brainstem lesions		MOG-IgG positive	Generally favorable recovery after immunotherapy	

## Neuroanatomical basis of the hiccup reflex

The hiccup reflex arc comprises afferent, central, and efferent components [1, 2]. The afferent limb includes sensory fibers from the vagus nerve, phrenic nerve, and thoracic sympathetic chain (T6–T12). These transmit visceral and somatic inputs to medullary centers. The central processing unit is localized within the dorsolateral medulla, including the nucleus tractus solitarius, nucleus ambiguus, reticular formation, and adjacent respiratory pattern generators. Additional modulatory input from hypothalamic centers and cervical spinal segments (C3–C5) further supports integration between autonomic and respiratory control networks [2, 7].

The efferent limb consists primarily of the phrenic nerve (C3–C5), intercostal nerves, and the recurrent laryngeal branch of the vagus nerve [1]. Lesions affecting dorsal medullary inhibitory circuits may result in disinhibition of this reflex, producing persistent hiccups.

Functional neuroimaging and lesion studies implicate the area postrema and adjacent nucleus tractus solitarius as key integrative centers [7].

## Immunopathogenesis of NMOSD and selective vulnerability of the area postrema

NMOSD is an autoimmune astrocytopathy mediated by AQP4-IgG antibodies targeting astrocytic water channels [8]. AQP4 is highly expressed in perivascular astrocytic foot processes and is particularly concentrated in circumventricular organs such as the area postrema.

The area postrema lacks a functional blood–brain barrier, allowing circulating AQP4-IgG to bind directly to astrocytes [9]. Antibody binding activates complement, leading to astrocyte destruction, secondary demyelination, and neuronal injury [8].

This anatomical and immunological susceptibility explains why intractable nausea, vomiting, and hiccups may precede optic neuritis or myelitis by weeks to months [10].

## Area postrema syndrome (APS)

### Diagnostic criteria

The 2015 International Consensus Criteria define APS as otherwise unexplained nausea, vomiting, or hiccups lasting ≥ 48 h, associated with dorsal medullary lesions.

In AQP4-IgG–positive patients, a single core clinical characteristic suffices for NMOSD diagnosis [3].

### Epidemiology

APS occurs in approximately 10–30% of NMOSD patients [10, 11]. In some cohorts, APS was the inaugural manifestation in up to 12–15% of cases. Patients harboring dorsal medullary lesions involving the area postrema demonstrate a markedly increased likelihood of intractable nausea and vomiting compared with those without such lesions [10].

Emerging cohort data indicate that patients presenting with APS as the initial manifestation of NMOSD may exhibit distinct relapse patterns. In recent multicenter analyses, APS-onset NMOSD was associated with a substantial risk of subsequent optic neuritis or longitudinally extensive transverse myelitis within the first two years of follow-up [12]. These findings suggest that APS should not be regarded as a benign or isolated brainstem event, but rather as a potential harbinger of more disabling relapses.

### Radiological features

MRI typically demonstrates T2-FLAIR hyperintensity in the dorsal medulla at the floor of the fourth ventricle. Lesions are often longitudinal and may enhance with gadolinium [4].

Unlike MS, lesions in NMOSD frequently extend contiguously and are centrally located [13]. Importantly, early MRI may occasionally be unremarkable despite active medullary dysfunction. Notably, advanced imaging studies suggest that subtle dorsal medullary inflammation may be radiologically occult on conventional MRI sequences. High-resolution imaging and dedicated thin-slice brainstem protocols have been proposed to improve detection sensitivity in suspected APS cases [14]. A recent 2025 report described a patient with APS and recurrent syncope in whom initial neuroimaging failed to reveal overt dorsal medullary abnormalities, while subsequent evaluation confirmed AQP4-IgG–positive NMOSD [15]. These findings emphasize that negative early imaging does not exclude APS in clinically suggestive cases. Nevertheless, current evidence is limited mainly to isolated case reports and small case series, and the true prevalence and diagnostic implications of MRI-negative APS remain uncertain.

### Clinical importance

Failure to recognize APS may lead to misdiagnosis as gastrointestinal disease, delaying immunotherapy [10]. Early high-dose intravenous methylprednisolone (IVMP) reduces inflammatory edema and often results in rapid symptom resolution [8].

## Differentiation from multiple sclerosis

MS is characterized by immune-mediated demyelination with oligoclonal IgG bands in cerebrospinal fluid in > 85% of cases [16].

Brainstem involvement occurs in MS but typically presents with internuclear ophthalmoplegia or ataxia rather than isolated hiccups. Although brainstem involvement may occur in multiple sclerosis (MS), selective dorsal medullary involvement resembling APS is distinctly uncommon [9, 13]. This difference likely reflects divergent immunopathological targets. In NMOSD, AQP4-IgG–mediated astrocytopathy preferentially affects regions with high aquaporin-4 expression and impaired blood–brain barrier integrity, such as the area postrema [9]. In contrast, MS primarily targets oligodendrocytes and exhibits a perivenular inflammatory pattern with characteristic ovoid, periventricular plaques (“Dawson’s fingers”) [13, 16]. The dorsal medulla does not represent a region of selective vulnerability in MS, which may explain the relative rarity of isolated persistent hiccups as an initial manifestation [13].

The predilection of NMOSD lesions for the dorsal medulla correlates anatomically with emetic reflex circuitry, a distribution pattern rarely observed in multiple sclerosis. MS lesions are ovoid, periventricular, and peripherally distributed [13, 16].

Unlike NMOSD, MS pathology primarily targets oligodendrocytes rather than astrocytes [17]. Importantly, several MS disease-modifying therapies (e.g., interferon-β) may worsen NMOSD [18], underscoring the need for correct diagnosis. Although persistent hiccups are classically associated with NMOSD, multiple sclerosis may rarely present with dorsal medullary involvement mimicking APS. Recent evidence underscores this diagnostic pitfall. A 2025 case report described a young woman presenting with refractory hiccups and dorsal medullary T2 hyperintensity who was ultimately diagnosed with multiple sclerosis according to the 2017 McDonald criteria after negative AQP4-IgG and MOG-IgG testing [19]. This observation highlights that isolated APS-like presentations are not pathognomonic for NMOSD and require careful clinicoradiological and serological correlation.

## Comparison with MOG antibody–associated disease (MOGAD)

MOGAD is defined by antibodies against myelin oligodendrocyte glycoprotein [20].

APS can occur but is less frequent than in NMOSD. Brainstem lesions in MOGAD are often more diffuse and may involve the pons or cerebellum.

Unlike NMOSD, isolated APS without optic neuritis or myelitis is rare in MOGAD. In contrast to NMOSD, in which APS may occur as an isolated attack in a substantial proportion of patients, MOGAD-related APS almost invariably accompanies or precedes additional demyelinating events [21]. Serological testing for MOG-IgG is therefore recommended when AQP4-IgG is negative [20]. Although APS is far more characteristic of NMOSD, comparative analyses demonstrate that MOGAD may rarely present with similar symptoms. However, lesion distribution patterns and recurrence profiles differ significantly, with MOGAD typically showing less selective dorsal medullary involvement and a lower frequency of isolated APS presentations [21]. These distinctions are clinically relevant when AQP4-IgG testing is negative. A broader comparison of the clinical, radiological, and immunopathological characteristics of NMOSD, MS, and MOGAD in patients presenting with persistent hiccups or APS is summarized in Table 2.

**Table 2:** Comparative Clinical, Radiological, and Immunopathological Features of NMOSD, MS, and MOGAD in Patients Presenting with Persistent Hiccups or APS

Feature	NMOSD	MS	MOGAD
Typical antibody	AQP4-IgG positive	None specific	MOG-IgG positive
Pathological target	Astrocytes (AQP4)	Oligodendrocytes	Myelin sheath (MOG)
APS frequency	Common (10–30%)	Rare	Uncommon
Isolated APS presentation	Possible and well-described	Rare, but reported	Very rare
MRI lesion location	Dorsal medulla, central, longitudinal	Periventricular, ovoid	Brainstem diffuse, pons/cerebellum
CSF oligoclonal bands	Usually negative	> 85% positive	Usually negative
Response to MS DMTs	May worsen	Beneficial	Variable
Risk of severe relapse	High	Moderate	Often steroid-responsive
Optic neuritis	Severe bilateral	Usually unilateral	Often bilateral
Myelitis	LETM	Short segment	LETM/conus
OCB positivity	15–30%	> 85%	10–20%
CSF pleocytosis	Frequent	Mild	Frequent
MRI brain pattern	Area postrema	Dawson fingers	Fluffy lesions
Enhancement pattern	Cloud-like	Ring/open ring	Patchy
Conus involvement	Rare	Rare	Common
Cortical lesions	Rare	Rare	Can occur
Response to interferon-beta	May worsen	Beneficial	Uncertain

### APS-like presentations beyond NMOSD

Although APS is considered highly characteristic of NMOSD, similar clinical presentations have occasionally been reported in other autoimmune inflammatory disorders. Recognition of these entities is important to avoid diagnostic delay and inappropriate treatment [5, 22].

#### Myelin oligodendrocyte glycoprotein antibody-associated disease (MOGAD)

APS-like manifestations are uncommon in MOGAD compared with AQP4-IgG-positive NMOSD [22, 23].

When present, persistent hiccups, nausea, and vomiting are usually accompanied by optic neuritis, transverse myelitis, or other brainstem syndromes rather than occurring as isolated manifestations [22].

Brain MRI may demonstrate brainstem lesions, although area postrema involvement appears to be considerably less frequent than in NMOSD [23].

Therefore, MOG-IgG testing should be considered in seronegative patients with suspected inflammatory brainstem disease [22].

Isolated APS presentations in MOGAD remain exceedingly rare and are supported mainly by case reports and small observational studies [23].

### Autoimmune GFAP astrocytopathy

Autoimmune glial fibrillary acidic protein (GFAP) astrocytopathy is another inflammatory disorder that may mimic APS [24, 25].

Patients typically present with meningoencephalitis, fever, headache, encephalopathy, or myelitis, although nausea, vomiting, and persistent hiccups have occasionally been described [24].

Characteristic MRI findings include linear radial perivascular enhancement extending from the ventricles, while cerebrospinal fluid analysis frequently reveals pleocytosis and elevated protein concentrations [24, 25].

Detection of GFAP-IgG, particularly in cerebrospinal fluid, supports the diagnosis [24].

Recognition of these distinctive radiological and laboratory features is helpful in differentiating GFAP astrocytopathy from NMOSD [25].

### Systemic autoimmune disorders

APS-like symptoms have also been reported in association with systemic autoimmune diseases such as systemic lupus erythematosus (SLE) and Sjögren syndrome [3, 5].

In these settings, clinicians should carefully evaluate for secondary NMOSD, as AQP4-IgG positivity may coexist with systemic autoimmune disorders [3].

Thorough serological evaluation and exclusion of alternative inflammatory causes are therefore essential when persistent hiccups and vomiting occur in patients with established systemic autoimmunity [5].

## Other neurological causes of intractable hiccups

Importantly, APS is not entirely specific to NMOSD. Similar clinical syndromes have been described in autoimmune glial fibrillary acidic protein (GFAP) astrocytopathy and systemic autoimmune conditions such as systemic lupus erythematosus [26, 27]. Recognition of these mimics is essential to avoid premature diagnostic closure and to guide appropriate immunotherapy. The major neurological causes of intractable hiccups together with their neuroanatomical correlations and characteristic clinical clues are summarized in Table 3.

### Stroke

Lateral medullary infarction (Wallenberg syndrome) is the most common structural cause [28]. Supratentorial strokes involving the insular cortex have also been implicated. Right hemispheric predominance in supratentorial cases suggests involvement of autonomic inhibitory networks, particularly within the insular cortex [29].

### Tumors

Brainstem gliomas and cavernomas may present with isolated persistent hiccups [30].

### Infections

Brainstem encephalitis, including varicella-zoster virus and tuberculosis, can involve the dorsal medulla [31].

### Autonomic and cardiac manifestations of dorsal medullary lesions

The nucleus tractus solitarius and neighboring medullary autonomic centers play a central role in cardiovascular reflex regulation. Inflammatory involvement of this region may therefore result not only in intractable hiccups but also in clinically significant autonomic disturbances, including bradyarrhythmias, sinus pauses, and syncope. Recent case reports and series have described reversible sick sinus syndrome and recurrent syncope in AQP4-IgG–positive NMOSD patients presenting with dorsal medullary inflammation consistent with APS [15]. These findings highlight the functional and anatomical proximity between the area postrema and cardiovascular regulatory nuclei, suggesting that APS may represent a broader medullary autonomic syndrome rather than an isolated emetic disorder. Accordingly, cardiac rhythm monitoring should be considered in selected patients with suspected brainstem involvement. These observations should be interpreted cautiously because available evidence is based predominantly on small case series and anecdotal reports.

**Table 3:** Causes of Intractable Hiccups with Neuroanatomical Correlation

Etiology	Anatomical Location	Mechanism	Key Clinical Clue
NMOSD	Area postrema	Astrocytic injury	APS triad
MS	Brainstem plaques	Demyelination	Other MS signs
MOGAD	Diffuse brainstem	Inflammatory demyelination	Optic neuritis history
Lateral medullary stroke	PICA territory	Disruption of NTS	Acute onset
Brainstem tumor	Pons/medulla	Mass effect	Progressive deficits
Encephalitis	Dorsal medulla	Inflammatory edema	Fever/CSF pleocytosis

## Therapeutic strategies

### Acute immunotherapy

IV methylprednisolone (1 g/day for 3–5 days) is first-line in NMOSD relapses [8]. Plasma exchange is recommended for steroid-refractory cases [32]. However, prolonged or repeated corticosteroid exposure carries risks including avascular necrosis, mandating careful risk–benefit assessment [2]. Complement inhibition may also play a role in selected severe acute presentations. In a recent 2025 case of NMOSD presenting with APS and cardiac conduction abnormalities, early administration of eculizumab was associated with rapid clinical stabilization [15]. However, evidence supporting the use of acute complement inhibition in this setting remains limited and derives largely from individual case reports rather than controlled studies.

### Long-term NMOSD therapy

Rituximab, eculizumab, satralizumab, and inebilizumab have demonstrated efficacy in relapse prevention [33–35].

### Symptomatic treatment of hiccups

Chlorpromazine is the only FDA-approved drug for hiccups [1]. Baclofen and gabapentin are frequently effective [36]. Refractory cases may benefit from cervical epidural block targeting C3–C5 segments [37].

## Diagnostic algorithm

**Fig. 2:**
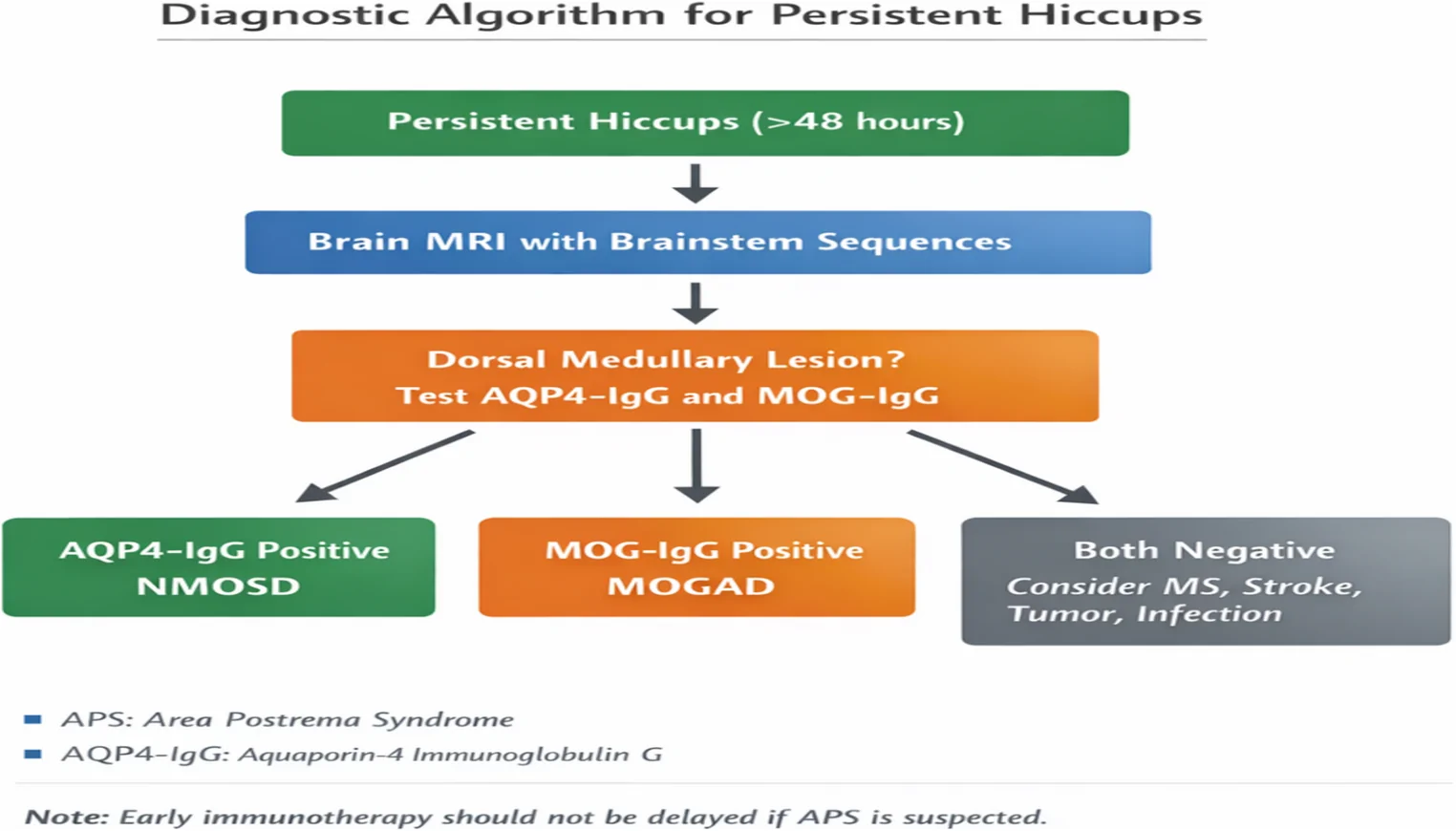
Proposed diagnostic algorithm for persistent and intractable hiccups, emphasizing early identification of NMOSD-related APS and differentiation from alternative neurological etiologies

Patients presenting with persistent hiccups lasting more than 48 h should undergo brain MRI with dedicated brainstem sequences. If a dorsal medullary lesion—particularly involving the area postrema—is identified, serologic testing for AQP4-IgG should be performed. AQP4-IgG positivity supports the diagnosis of NMOSD. If AQP4-IgG is negative, testing for MOG-IgG is recommended to evaluate for MOGAD. In cases where both antibodies are negative, alternative etiologies—including multiple sclerosis, vascular lesions, neoplasms, and infections—should be systematically considered. Early immunotherapy may be considered in clinically suspected APS, even if initial imaging findings are inconclusive [3, 15, 20]. In patients with strong clinical suspicion of APS, negative initial MRI findings should not exclude NMOSD. Repeat imaging, thin-slice brainstem sequences, and evaluation for autoimmune mimics including MOGAD and GFAP astrocytopathy should be considered (Fig. 2).

The diagnostic algorithm is based on the 2015 International Consensus Criteria for NMOSD [3], current recommendations regarding MOG-IgG testing [14], and recent clinical observations highlighting MRI-negative APS presentations [27].

## Future directions

Despite growing recognition of APS as a core manifestation of NMOSD, several critical gaps remain in our understanding of persistent hiccups as a brainstem red flag.

First, prospective multicenter registries focusing specifically on APS and other dorsal medullary syndromes are needed. Most available data derive from retrospective cohorts or isolated case reports, limiting precise estimation of incidence, relapse risk, and long-term neurological outcomes. Structured prospective data collection would allow better characterization of patients presenting initially with intractable hiccups and clarify the proportion who subsequently develop optic neuritis or longitudinally extensive transverse myelitis [38, 39].

Second, biomarker development represents a major unmet need. While AQP4-IgG and MOG-IgG testing have transformed diagnostic accuracy, additional biomarkers reflecting astrocytic injury may improve early detection of subclinical brainstem inflammation. Serum GFAP has emerged as a promising marker of disease activity in NMOSD and correlates with astrocytic damage and relapse severity [40, 41]. Longitudinal biomarker profiling during APS attacks may help define temporal relationships between antibody titers, complement activation, and clinical symptom onset. Recent investigations have demonstrated that serum GFAP levels correlate with astrocytic injury and lesion burden in AQP4-IgG–positive NMOSD, particularly during acute attacks [42, 43]. In contrast, neurofilament light chain (NfL) appears to reflect axonal injury rather than primary astrocytopathy. Longitudinal biomarker profiling during APS episodes may therefore provide insights into the temporal dynamics of astrocytic damage and relapse risk.

Third, advanced neuroimaging techniques warrant systematic evaluation. High-resolution 3 T or ultra–high-field 7 T MRI, thin-slice brainstem FLAIR imaging, diffusion tensor imaging, and quantitative susceptibility mapping may enhance detection of subtle dorsal medullary lesions that remain occult on conventional sequences [44, 45]. Artificial intelligence–assisted image analysis could further improve sensitivity for early inflammatory changes in small brainstem structures.

Fourth, the autonomic consequences of dorsal medullary inflammation require deeper exploration. The nucleus tractus solitarius and adjacent medullary autonomic centers regulate cardiovascular reflexes, yet systematic autonomic testing in NMOSD remains limited. Prospective evaluation using heart rate variability analysis, tilt-table testing, and continuous cardiac rhythm monitoring may clarify the prevalence and prognostic relevance of bradyarrhythmias and syncope in inflammatory brainstem disease [46, 47].

Finally, optimal therapeutic timing remains incompletely defined. Although early immunotherapy appears beneficial in clinically suspected APS, controlled data evaluating treatment initiation prior to radiological confirmation are lacking. Observational studies and adaptive trial designs are needed to determine whether very early intervention modifies long-term relapse trajectories and disability accumulation [48, 49].

Collectively, these research priorities underscore the need to reconceptualize persistent hiccups not merely as a benign reflex phenomenon, but as a potential sentinel event of brainstem immunopathology.

## Limitations

This review has several limitations. First, the available literature is largely composed of case reports and small observational cohorts, limiting the strength of evidence. Second, heterogeneity in imaging protocols and antibody testing methodologies may affect comparability across studies. Third, publication bias may favor reporting of severe or atypical presentations such as intractable hiccups. Finally, the narrative design precludes quantitative estimation of incidence or treatment effect sizes.

## Conclusion

Intractable hiccups are not merely benign nuisances but may represent an early brainstem manifestation of demyelinating disease. NMOSD, particularly through APS, is the prototypical etiology. Recent 2025 reports further expand the clinical spectrum of brainstem red flags, demonstrating that both multiple sclerosis and NMOSD may initially manifest with refractory hiccups, occasionally accompanied by autonomic instability.Prompt recognition, antibody testing, and immunotherapy are critical to preventing irreversible disability. Neurologists must maintain a high index of suspicion when encountering unexplained persistent hiccups.
